# Perception about the Major Health Challenges in Different Swine Production Stages in Spain

**DOI:** 10.3390/vetsci11020084

**Published:** 2024-02-10

**Authors:** Alba Meléndez, María Teresa Tejedor, Olga Mitjana, María Victoria Falceto, Laura Garza-Moreno

**Affiliations:** 1 Department of Animal Pathology, University of Zaragoza, 50013 Zaragoza, Spain; 701525@unizar.es; 2 Department of Anatomy, Embriology and Animal Genetics, Faculty of Veterinary Sciences, University of Zaragoza, 50013 Zaragoza, Spain; ttejedor@unizar.es; 3 Agroalimentary Institute of Aragon-IA2, Department of Animal Pathology, Universidad de Zaragoza-CITA, 50009 Zaragoza, Spain; omitjana@unizar.es (O.M.); vfalceto@unizar.es (M.V.F.); 4 Ceva Salud Animal, 08028 Barcelona, Spain

**Keywords:** swine, health challenge, production, questionnaire, Spain

## Abstract

**Simple Summary:**

The Spanish swine sector has experienced continuous growth during recent years, becoming the largest pig sector in Europe and one of the largest exporters of pig meat worldwide. One of the main challenges for the stability and productivity of the Spanish swine industry is the presence of several swine diseases, resulting in an important economic impact. Information on the current frequency and importance of the main swine diseases which affect the Spanish pig industry in each phase could help in optimizing and prioritizing the efforts within disease control programs. This study described the frequency and importance of different pathogens for veterinarians and consultants in the production phases, as well as the most used tools for controlling such pathogens and diseases.

**Abstract:**

One of the main challenges for the sustainability and productivity of the Spanish swine industry is health instability, resulting in significant economic losses. Information on the main swine diseases which affect the Spanish pig industry could help in optimizing the efforts within control programs. This study determined the frequency of occurrence of the main diseases in Spain and the main control tool used, based on perceptions from veterinarians and consultants in a specific survey. Results showed that *Streptococcus* (*S.*) *suis*, *E. coli,* and coccidia are the most frequent pathogens in the gestation and lactation phase, whereas the most important were Porcine Reproductive and Respiratory Syndrome virus (PRRSV). In the nursery phase, the most frequent were *S. suis*, *E. coli,* and PRRSV, the latter being the most important for the participants. Finally, in the fattening phase, PRRSV and *Actinobacillus pleuropneumoniae* were the most frequent and important pathogen, respectively. Statistical differences among responses were detected with respect to the location and the gestation and lactation phases by farm size. Regarding the tools used for controlling the diseases, vaccination was the main strategy in all production phases, except in the fattening period, in which antibiotherapy was the most common response from the participants. Finally, the improvement of management practices was the most proposed tool, suggesting its importance within control programs.

## 1. Introduction

Swine diseases generate important economic losses for the industry worldwide, associated with reduced performance, increased mortality, and treatment cost as well as a negative impact on welfare and public health. Overall, multiple pathogens are involved, and polymicrobial infections are usually present in such diseases [1].

Reproductive failure is one of the main causes of sow culling in the swine industry, often being difficult to diagnose, as reproductive problems are usually multifactorial [2]. Nowadays, one of the most important reproductive diseases is Porcine Reproductive and Respiratory Syndrome (PRRS). In fact, this syndrome is caused by PRRSV, and a recent study performed in Europe and North America estimated its cost to be USD 6.25–15.25/pig marketed [3,4,5].

The main respiratory diseases are caused by primary pathogens, viruses, and/or bacteria. Most of the time, such respiratory processes are complicated by other secondary pathogens. This combination of pathogens complicates the diagnosis and treatment of respiratory diseases and a global approach is required [6]. These respiratory diseases can affect all production phases. However, the nursery and fattening phases are more frequently affected because pigs are at higher densities than in previous stages. The combination of primary and secondary pathogens, viral and/or bacterial, is known as Porcine Respiratory Disease Complex (PRDC). This infectious process causes important economic losses to the swine industry, in terms of reduced performance, increased mortality, and treatment costs as well as the condemnation of carcasses at slaughter [7].

Regarding digestive diseases, they are also important in swine production, causing important economic losses. Multiple enteric pathogens are usually involved in digestive processes, resulting in complex clinical disease patterns and difficulties in arriving at successful control measures [8]. These digestive diseases can affect breeding farms (mainly the lactation period) and nursery phases, where the animals are still adapting to the environment and the different feeding pattern, being more susceptible to digestive problems [9]. However, fattening pigs can also be affected by diseases such as proliferative enteropathies and swine dysentery, among others [10,11]. In relation to digestive pathogens, *E. coli*, rotavirus, and/or coccidia are some of the most frequently observed in the lactation period [12].

Additionally, restrictions on the use of antimicrobials have led to the incidence of emerging pathogens such as *Glaeserella parasuis* (*G. parasuis*), and even zoonotic pathogens such as *Streptococcus suis* (*S. suis*) have increased during recent years [13]. In fact, it is important to verify the veterinarians’ perception of the presence (VPP) of those pathogens affecting the swine industry due to economic losses generated and the potential risk to public health. Therefore, this study aimed to describe the frequency and importance of the major challenges for the swine industry in the different production stages, based on perceptions from veterinarians and consultants, collected through a specific survey, performed in the Spanish swine sector.

## 2. Materials and Methods

### 2.1. Questionnaire Description and Distribution

The questionnaire was designed and divided for each swine production period [(1) gestation and lactation, (2) nursery, and (3) fattening]. In each period, the 20 most important pathogens were included, and the questions for each phase were as follows:

- General questions: Number of farms, number of sows (1) or animals (2), (3) and location.
- Frequency of occurrence of each pathogen: The responses correspond to the frequency with which the participants encountered the pathogens on all the farms they manage. It was measured with the 6-point Likert scale, although it does not correspond to the objective measure of frequency. A range of 0 to 5 was established to determine the frequency of occurrence of each pathogen/disease, with 1 being very infrequent, 5 very frequent, and 0 never observed.
- The most significant pathogen/disease: Among those selected with a 5.
- Most used tools for controlling these pathologies: Vaccination, antibiotic treatment, sacrifice, other.

As the questionnaires were divided into three parts corresponding to each phase, from each questionnaire answered by a single veterinarian, 1 to 3 responses could be obtained.

The questionnaire was created with the Google Forms platform. It was distributed using a link (https://docs.google.com/forms/d/e/1FAIpQLSdloAC1KcTzeDG6dGJ (accessed on 1 February 2024) among the swine veterinarians by WhatsApp and e-mail, fVlqDjWBGKyw83ocSFnU9p61r MS6Q/viewform?embedded=true) to the swine veterinarians of “Asociación Nacional de Veterinarios de Porcino” (ANAVEPOR). The questionnaire was answered voluntarily by field veterinarians and consulting veterinarians from the Spanish white pork industry. The collected data were the product of the voluntary participation of veterinarians and are reported in a descriptive fashion.

### 2.2. Data Analysis

Responses of each production phase were compared (1) between field practitioners and consultants and (2) between Aragon and Catalonia and the rest of Spain. Additionally, data from the gestation and lactation stages were compared according to farm categorization based on farm size following the criteria of the Porc d’or awards (1: 10–200; 2: 201–500; 3: 501–1000; 4: 1001–2000; 5: >2001) (http://www.bdporc.irta.es accessed on 1 November 2022).

Statistical analyses were performed using IBM SPSS version 26 software (SPSS, Chicago, IL, USA). For every considered pathology, crosstabs and chi square test for independence were used for comparing frequencies of responses between groups (consultants vs. field veterinarians, Aragon and Catalonia versus the rest of Spain, size of farms). Bonferroni correction was applied for multiple comparisons. Differences were considered statistically significant when the *p*-value was lower than 0.05 (*p* < 0.05).

## 3. Results

**Figure 1:**
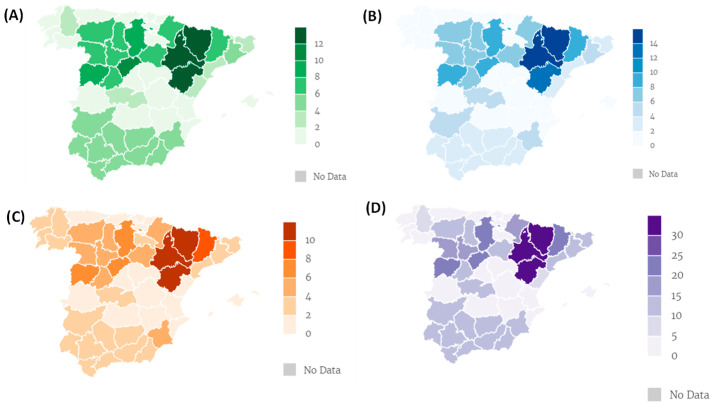
Distribution of participants by autonomous communities. (**A**) Gestation and lactation (GyL); (**B**) nursery (N); (**C**) fattening (F); (**D**) all phases.

A total of 95 veterinarians answered the questionnaire and 262 responses were obtained because they answered one, two, or all three phases of production, as appropriate. A total of 69 (72.6%) participants shared the farm localization, while 26 (27.4%) did not. Of the former, 146 responses (55.7%) belonged to Aragon and Catalonia, while 116 (44.3%) belonged to the rest of Spain (Figure 1).

### 3.1. Gestation and Lactation

This phase refers to pregnant and lactating sows, as well as suckling piglets. It was answered by 66 field veterinarians and 11 consultants. Aragon is the autonomous community with the most responses from veterinarians, followed by Castilla and Leon, especially the province of Segovia (Figure 1).

#### 3.1.1. Frequency of the Pathologies

For field veterinarians, the perception regarding the most frequent pathologies during the gestation and lactation phase were *S. suis* (10/66; 15.2%), *E. coli* (10/66; 15.2%), and coccidia (8/66; 12.1%) (Table 1). In the case of consultants, *S. suis* (3/11; 27.3%), *E. coli* (3/11; 27.3%), and *C. perfringens type A* (3/11; 27.3%) were selected as the most frequent pathogens (Table 2). There were not significant differences regarding these pathologies between consultants and field veterinarians’ responses, except for *C. perfringens type A*. Considering the autonomous communities, significant differences were detected between Aragon and Catalonia with respect to the rest of Spain in terms of the VPP for swine erysipelas, among others (Table 3). According to farm size, significant differences were detected in different pathologies, as detailed in Table 3, e.g., a difference was detected in Rotavirus.

#### 3.1.2. Importance of the Pathologies According to Responses of Participants

Field veterinarians chose PRRSV (50.7%), *S. suis* (18.3%), and *E. coli* (12.7%) as most significant and consultants selected the same pathogens, but they considered different VPP among PRRSV (50.0%), *E. coli* (20.0%), and *S. suis* (20.0%). Significant differences between the responses of field veterinarians and consultants were detected in the VPP of Rotavirus and in the responses according to farm size, mainly for PRRS.

#### 3.1.3. Most Used Tools

The tools selected as the most used by field veterinarians were vaccination (48.6%), antibiotherapy (24.6%), and culling (8.6%). In the case of consultants, they also indicated vaccination (47.4%) as the first option, followed by antibiotherapy (15.8%). Considering the autonomous communities of Spain, significant differences in the use of vaccines were detected among them, with them being less used in Aragon and Catalonia. Considering the farm size, significant differences were also observed in the use of vaccines and sacrifice.

### 3.2. Nursery

This phase was reported by 65 field veterinarians and 12 consultants. Aragon is again the autonomous community with the highest number of responses, especially in the provinces of Huesca and Zaragoza, followed by Castilla and Leon, and Catalonia (Figure 1).

#### 3.2.1. Frequency of the Pathologies

Field veterinarians and consultants agreed that the perception of the most frequent pathologies included *S. suis* (26/65; 40.0% and 5/12; 41.7%, respectively), *E. coli* (14/65; 21.5% and 4/12; 33.3%, respectively), and PRRSV (13/65; 20.0% and 1/12; 8.3%, respectively) (Table 1) (Table 2). No significant differences were found for these pathologies between consultants and veterinarians; however, consultants and veterinarians significantly differed for *M. hyopneumoniae*, PED, *C. perfringens type A*, *C. difficile*, among others. Significant differences were found for SIV, *L. intracellularis,* and PED between communities (Table 4).

#### 3.2.2. Importance of the Pathologies According to Responses of Participants

Field veterinarians considered the most significant pathology to be PRRSV (33.3%), followed by *S. suis* (26.4%) and *E. coli* (26.4%), while for consultants, the most significant was *S. suis* (50.0%).

#### 3.2.3. Most Used Tools

The most frequently used tools according to field veterinarians and consultants were antibiotherapy (38.0% and 33.3%, respectively) and vaccination (33.9% and 29.2%, respectively). Significative differences were not detected for the use of tools between field veterinarians and consultants or between communities.

### 3.3. Fattening

This phase was answered by 54 field veterinarians and 12 consultants. Aragon, as in the two previous phases, is the autonomous community with the highest number of responses. The province of Lleida is in second position, followed by some provinces of Castilla and Leon. Murcia gains weight in this phase with respect to the previous ones (Figure 1).

#### 3.3.1. Frequency of the Pathologies

Field veterinarians considered that the most frequent pathologies, based on their perceptions, were PRRSV (5/54; 8.8%), *L. intracellularis* (5/54; 8.8%), and *App* (5/54; 8.8%) (Table 1). For the consultants, they were App (2/12; 16.7%) followed by *L. intracellularis* (1/12; 8.3%) and PRRSV (1/12; 8.3%) (Table 2). Differences between the the responses of veterinarians and consultants were statistically significant for *C. perfringens type C* (Table 5). Significant differences were found for SIV, *L. intracellularis*, *G. parasuis,* and PED between communities (Table 5).

#### 3.3.2. Importance of the Pathologies According to Responses of Participants

Field veterinarians and consultants chose *App* (29.5% and 46.7%, respectively) followed by PRRSV (18.0% and 20.0%, respectively) as the most significant ones.

#### 3.3.3. Most Used Tools

Both consultants and field veterinarians selected vaccination (42.7% and 42.9%, respectively) as the most used tool, followed by antibiotherapy (39.8% and 38.1%, respectively). Management is the most frequently used tool, with a significantly higher frequency in Aragon and Catalonia than in the rest of Spain.

## 4. Discussion

Swine diseases are an important health challenge, and these can generate substantial economic losses in the pig industry. Most often, multiple infectious agents (primary and/or secondary) are involved in those processes, requiring a global approach for controlling the disease. In order to describe the current importance of the different pathogens in the common processes observed in the field, this study aimed to determine the frequency and importance of the main infectious diseases observed in each production phase by a questionnaire answered by field veterinarians and consultants.

Obtained responses were compared considering the production phase, as the importance of pathogens affecting different production phases might differ. Likewise, analyses of responses were also performed considering the location. In fact, answers were divided by the autonomous communities into two regions: Aragon and Catalonia and the rest of Spain. These two regions were compared to the rest of Spain because Aragon (26.8%) and Catalonia (24.3%) represent more than half of the pig census in Spain [14].

In the gestation and lactation period, S. suis and E. coli were selected as the most frequent pathologies. Nevertheless, both field veterinarians and consultants identified PRRS as the most important disease. The importance of PRRS is explained by the great economic losses it causes to the sector, generating an increase in production costs [4,15]. The main problems of control programs against this virus are the persistence of negative subpopulations, in which the virus can recirculate, as well as the high rate of variability and mutability of the different virus strains, even its capability of recombination among them [16,17]. In addition, several studies demonstrated that different PRRSV strains do not show cross-immunity among them [18,19]. Considering the autonomous communities, the frequency of swine erysipelas was lower in Aragon and Catalonia when compared to the rest of Spain. This fact might be explained because this disease does not manifest itself continuously, although most animals can be carriers and only show clinical signs in situations of immunosuppression [20,21]. In relation to farm size, differences were observed for almost all pathologies, as expected, since management is more complicated in larger farms compared to smaller ones, as well as the control and eradication of pathogens. Regarding tools used for controlling diseases, commercial vaccines were the most used ones according to participant responses. Interestingly, the frequency of the use of vaccination was lower in Aragon and Catalonia compared to the rest of Spain. These results might be explained since some pathologies differ in frequency between the two territories, being less present in Aragon and Catalonia. Nevertheless, other strategies (i.e., management practices, biosecurity measures, etc.) should be also considered and further studies are needed to evaluate its impact.

Regarding nursery period, *E. coli* and *S. suis* are the most frequent diseases selected. Nevertheless, some agents, such as PRRS, increase in frequency and in importance, according to responses from field veterinarians. In the case of consultants, they chose *S. suis* as the most important. This finding could be explained because the bacterium at this stage plays a very important role as a secondary pathogen [5,22]. In fact, most weaned piglets are infected, being carriers [23,24], and the bacterium takes advantage of primary infections such as PRRS or *M. hyopneumoniae* to proliferate and generate clinical signs [13,22]. Most of the pathologies had a high frequency of occurrence in Aragon and Catalonia, except *L. intracellularis*, where it was framed as infrequent in these territories. This finding may suggest that in these territories, the bacterium is more controlled compared to the rest of Spain.

A change in the tendency of the most frequent pathologies is observed in the fattening period, except for PRRS, which remains within the three most frequent pathologies. In this case, field veterinarians and consultants considered that the most significant pathology at this stage was *App*. This finding could be related to the important economic losses associated with this bacterium, due to the high mortality and the worsening of productive parameters [25]. In addition, the costs derived from the control and treatment of pleuropneumonia caused by *App* are added to the economic losses at the slaughterhouse, due to the seizure of carcasses because of the presence of pleuritis [26,27]. Regarding the importance of *L. intracellularis* in fattening, it has greatly increased its prevalence in Spain in recent years. This bacterium is sensitive to many antibiotics and there are commercial vaccines to minimize its economic impact [28,29]. Its persistence in the environment makes its eradication very complicated once it has entered a farm [10,28]. Considering the differences between communities, most respiratory pathogens had a greater presence in Aragon and Catalonia than in the rest of Spain. It is important to note that most of these pathogens are airborne viruses and their dissemination could be facilitated by high-density areas, such as where the farms are [30]. Indeed, a recent study performed by VanderWaal and Deen [7] suggested that current control and biosecurity measures are effective against bacteria such as *App*, but that in the case of airborne viruses, they are not fully effective if the intensification is high.

One of the main limitations of this study is that the prevalence and incidence data of pathogens on farms are not available, so the frequency has been determined subjectively based on the VPP of each pathogen on farms. On the other hand, the presence of pathogens differs between farms, which could influence veterinarians’ responses. The present study was based on a questionnaire administered through electronic platforms and the veterinarians were free to adhere or not. In such situations, a participation bias could arise if those who respond to the survey differ in the outcome variable. Since the participants were completely anonymous, we could not establish whether there were differences between respondents and non-respondents that could affect their responses. Also, it is impossible to know the exact response rate, since the questionnaire was sent by various means and its distribution requested. Therefore, the exact number of people who received the questionnaire is unknown. Given that this bias is very difficult to control in this type of survey, we tried to reach the maximum number of professionals, with the hope of obtaining a greater number of responses; the greater the number of responses, the greater the representativeness of the sample analyzed and the fewer the problems associated with this bias. However, it should be noted that this is another limitation of this study.

## 5. Conclusions

The present study showed that PRRS is one of the most frequent and important concerns for swine veterinarians and consultants in Spanish breeding and nursery farms, while *App* is considered as the most important in the fattening period. Likewise, the frequency of occurrence and importance of different diseases was dependent on farm size and farm location area. Moreover, vaccination strategies were selected as the most used tool for controlling diseases, although its use varied among areas. Further studies should be conducted in order to evaluate the impact of the different control strategies on health stability in each production stage.

## Figures and Tables

**Table 1:** Results of frequency of the different pathogens associated with respiratory, digestive, reproductive, and systemic diseases evaluated in this study according to field veterinarian responses (%).

Group	Infectious Agent/Pathology	0			1			2			3			4			5			N/R		
		G-L	N	F	G-L	N	F	G-L	N	F	G-L	N	F	G-L	N	F	G-L	N	F	G-L	N	F
Reproductive	PRRSV	13.6	7.7	7.0	7.6	4.6	17.5	18.2	16.9	22.8	25.8	32.3	24.6	21.2	18.5	17.5	10.6	20.0	8.8	3.0	0.0	1.8
Respiratory	SIV	19.7	20.0	17.5	36.4	32.3	19.3	18.2	13.8	22.8	18.2	18.5	24.6	6.1	13.8	14.0	0.0	0.0	0.0	1.5	1.5	1.8
	*M. hyopneumoniae*	21.2	13.8	7.0	34.8	32.3	10.5	10.6	21.5	22.8	15.2	20.0	29.8	12.1	9.2	22.8	4.5	1.5	5.3	1.5	1.5	1.8
	*P. multocida*	19.7	16.9	7.0	45.5	27.7	26.3	16.7	30.8	28.1	10.6	15.4	19.3	7.6	7.7	17.5	0.0	1.5	0.0	0.0	0.0	1.8
	*App*	28.8	23.1	8.8	36.4	35.4	15.8	15.2	18.5	24.6	13.6	15.4	17.5	4.5	6.2	24.6	1.5	1.5	8.8	0.0	0.0	0.0
	*B. bronchiseptica*	33.3	23.1	45.6	36.4	38.5	22.8	15.2	21.5	19.3	6.1	4.6	8.8	3.0	6.2	1.8	3.0	1.5	0.0	3.0	4.6	1.8
Septicemic	*G. parasuis*	28.8	6.2	22.8	25.5	10.8	35.1	24.2	21.5	24.6	10.6	29.2	10.5	9.1	20.0	5.3	0.0	12.3	0.0	1.5	0.0	1.8
	*S. suis*	7.6	0.0	8.8	13.6	4.6	29.8	22.7	7.7	26.3	19.7	21.5	17.5	19.7	23.1	10.5	15.2	40.0	5.3	1.5	3.1	1.8
	*E. rhusiopathiae*	42.4	66.2	42.1	37.9	26.2	29.8	10.6	6.2	14.0	7.6	0.0	7.0	1.5	0.0	5.3	0.0	0.0	1.8	0.0	1.5	0.0
Digestive	PCV2	24.2	9.2	15.8	40.9	43.1	45.6	16.7	16.9	19.3	6.1	18.5	8.8	10.6	4.6	8.8	1.5	6.2	1.8	0.0	1.5	0.0
	*L. intracellularis*	36.4	40.0	10.5	36.4	40.0	14.0	7.6	9.2	29.8	6.1	4.6	14.0	9.1	4.6	21.2	3.0	1.5	8.8	1.5	0.0	1.8
	TGE (Transmissible Gastroenteritis)	66.7	64.6	64.9	24.2	24.6	24.6	3.0	4.6	3.5	0.0	3.1	0.0	0.0	0.0	0.0	0.0	0.0	0.0	6.1	3.1	7.0
	PED (Porcine Epidemic Diarrhea)	34.8	35.4	47.4	33.3	35.4	22.8	18.2	12.3	24.6	7.6	9.2	1.8	1.5	1.5	1.8	3.0	3.1	0.0	1.5	3.1	1.8
	*E. coli*	4.5	1.5	14.0	6.1	4.6	31.6	28.8	15.4	28.1	10.6	14.6	21.1	34.8	32.3	1.8	15.2	21.5	1.8	0.0	0.0	1.8
	*C. perfringens type A*	15.2	46.2	45.6	31.8	32.3	28.1	24.2	13.8	12.3	22.7	3.1	5.3	4.5	1.5	3.5	1.5	0.0	0.0	0.0	3.1	5.3
	*C. perfringens type C*	18.2	44.6	38.6	19.7	32.3	33.3	30.3	12.3	14.0	15.2	4.6	8.8	6.1	1.5	1.8	3.0	0.0	0.0	7.6	4.6	3.5
	Coccidia	9.1	72.3	77.2	30.3	20.0	14.0	25.8	4.6	3.5	16.7	1.5	0.0	4.5	0.0	0.0	12.1	0.0	0.0	1.5	1.5	5.3
	*C. difficile*	22.7	58.5	59.6	39.4	26.2	17.5	21.2	9.2	8.8	7.6	1.5	3.5	6.1	0.0	0.0	0.0	0.0	1.8	3.0	4.6	8.8
	Rotavirus	24.2	53.8	57.9	24.2	26.2	28.1	19.7	12.3	7.0	13.6	3.1	0.0	9.1	1.5	0.0	7.6	0.0	0.0	1.5	3.1	7.0
	Gastric ulcers	21.2	58.5	12.3	33.3	30.8	28.1	25.8	3.1	29.8	6.1	4.6	22.8	9.1	0.0	1.8	0.0	0.0	3.5	4.5	3.1	1.8

The frequency of occurrence of each pathogen/disease was measured on a 0–5 scale (1 very infrequent, 5 very frequent, and 0 never observed). N/A: No answer.

**Table 2:** Results of frequency of the different pathogens associated with respiratory, digestive, reproductive, and systemic diseases evaluated in this study according to consultant responses (%).

Group	Infectious Agent/Pathology	0			1			2			3			4			5			N/R		
		G-L	N	F	G-L	N	F	G-L	N	F	G-L	N	F	G-L	N	F	G-L	N	F	G-L	N	F
Reproductive	PRRSV	0.0	0.0	16.7	9.1	16.7	16.7	27.3	16.7	25.0	18.2	16.7	16.7	27.3	33.3	8.3	9.1	8.3	8.3	9.1	8.3	8.3
Respiratory	SIV	0.0	0.0	0.0	9.1	25.0	25.0	45.5	25.0	25.0	27.3	25.0	25.0	9.1	16.7	16.7	0.0	0.0	0.0	9.1	8.3	8.3
	*M. hyopneumoniae*	18.2	33.3	16.7	45.5	0.0	16.7	9.1	16.7	25.0	18.2	41.7	25.0	0.0	0.0	8.3	0.0	0.0	0.0	9.1	8.3	8.3
	*P. multocida*	18.2	25.0	8.3	36.4	16.7	33.3	18.2	16.7	25.0	18.2	8.3	8.3	0.0	25.0	16.7	0.0	0.0	0.0	9.1	8.3	8.3
	*App*	9.1	8.3	0.0	36.4	16.7	25.0	27.3	33.3	0.0	9.1	25.0	25.0	9.1	0.0	25.0	0.0	0.0	16.7	9.1	16.7	8.3
	*B. bronchiseptica*	27.3	25.0	33.3	27.3	16.7	33.3	9.1	41.7	16.7	9.1	8.3	0.0	18.2	0.0	0.0	0.0	0.0	0.0	9.1	8.3	16.7
Septicemic	*G. parasuis*	9.1	0.0	16.7	36.4	8.3	33.3	27.3	33.3	16.7	18.2	25.0	16.7	0.0	25.0	8.3	0.0	0.0	0.0	9.1	8.3	8.3
	*S. suis*	0.0	0.0	16.7	27.3	0.0	50.0	27.3	16.7	8.3	0.0	25.0	16.7	9.1	8.3	0.0	27.3	41.7	0.0	9.1	8.3	8.3
	*E. rhusiopathiae*	27.3	58.3	33.3	36.4	16.7	25.0	0.0	16.7	25.0	27.3	0.0	0.0	0.0	0.0	8.3	0.0	0.0	0.0	9.1	8.3	8.3
Digestive	*L. intracellularis*	36.4	33.3	0.0	36.4	8.3	8.3	9.1	33.3	50.0	9.1	8.3	16.7	0.0	0.0	8.3	0.0	0.0	8.3	9.1	16.7	8.3
	PCV2	9.1	16.7	8.3	36.4	33.3	41.7	18.2	25.0	25.0	18.2	8.3	8.3	9.1	8.3	8.3	0.0	0.0	0.0	9.1	8.3	8.3
	TGE (Transmissible Gastroenteritis)	54.5	50.0	58.3	18.2	8.3	25.0	0.0	25.0	8.3	9.1	8.3	0.0	9.1	0.0	0.0	0.0	0.0	0.0	9.1	8.3	8.3
	PED (Porcine Epidemic Diarrhea)	9.1	16.7	33.3	27.3	8.3	33.3	27.3	41.7	16.7	18.2	25.0	8.3	9.1	0.0	0.0	0.0	0.0	0.0	9.1	8.3	8.3
	*E. coli*	0.0	0.0	16.7	9.1	8.3	25.0	9.1	0.0	25.0	0.0	25.0	8.3	36.4	25.0	16.7	27.3	33.3	0.0	18.2	8.3	8.3
	*C. perfringens type A*	0.0	41.7	75.0	18.2	16.7	0.0	9.1	0.0	8.3	18.2	16.7	8.3	9.1	16.7	0.0	27.3	0.0	0.0	18.2	8.3	8.3
	*C. perfringens type C*	0.0	33.3	50.0	9.1	25.0	0.0	18.2	8.3	16.7	18.2	16.7	16.7	27.3	8.3	8.3	18.2	0.0	0.0	9.1	8.3	8.3
	Coccidia	0.0	58.3	83.3	45.5	16.7	8.3	0.0	8.3	0.0	9.1	8.3	0.0	18.2	0.0	0.0	18.2	0.0	0.0	9.1	8.3	8.3
	*C. difficile*	0.0	50.0	75.0	27.3	16.7	16.7	18.2	8.3	0.0	9.1	16.7	0.0	18.2	0.0	0.0	18.2	0.0	0.0	9.1	8.3	8.3
	Rotavirus	0.0	16.7	66.7	0.0	41.7	16.7	27.3	25.0	8.3	27.3	8.3	0.0	18.2	0.0	0.0	18.2	0.0	0.0	9.1	8.3	8.3
	Gastric ulcers	18.2	58.3	25.0	18.2	25.0	25.0	18.2	0.0	16.7	18.2	0.0	8.3	9.1	0.0	8.3	0.0	0.0	0.0	18.2	16.7	16.7

The frequency of occurrence of each pathogen/disease was measured on a 0–5 scale (1 very infrequent, 5 very frequent, and 0 never observed). N/A: No answer.

**Table 3:** Results from the variables used in the analysis about the frequency of occurrence of the main pathogens in the gestation and lactation phase (%).

Differences	Pathogen	Variable	0	1	2	3	4	5	Sign.
Between consultants and field veterinarians	SIV	Con.	0.0	10.0	50.0	30.0	10.0	0.0	0.045 *
		Vet.	17.0	40.4	17.0	21.3	4.3	0.0	
	TGE	Con.	60.0	20.0	0.0	10.0	10.0	0.0	0.022 *
		Vet.	70.8	21.3	5.6	2.2	0.0	0.0	
	*C. perfringens type A*	Con.	0.0	22.2	11.1	22.2	11.1	33.3	<0.001 **
		Vet.	11.7	35.1	20.2	28.7	3.2	1.1	
	*C. perfringens type C*	Con.	0.0	10.0	20.0	20.0	30.0	20.0	0.049 *
		Vet.	16.3	18.5	35.9	17.4	6.5	5.4	
	*C. difficile*	Con.	0.0	30.0	20.0	10.0	20.0	20.0	<0.001 **
		Vet.	17.6	40.7	29.7	7.7	4.4	0.0	
Between autonomous communities	*E. rhusiopathiae*	A and C	24.6	47.4	10.5	15.8	1.8	0.0	0.045 *
		Rest	45.9	45.9	8.1	0.0	0.0	0.0	
Between size of farms	Rotavirus	1	0.0	33.3	0.0	66.7	0.0	0.0	<0.001 **
		2	77.8	11.1	11.1	0.0	0.0	0.0	
		3	13.3	36.7	23.3	10.0	6.7	10.0	
		4	5.9	14.7	29.4	35.3	11.8	2.9	
		5	18.8	18.8	31.3	0.0	6.3	25.0	

Con.: Consultants; Vet.: Field veterinarians; Sign.: Significant differences (* *p*-value < 0.05; ** *p*-value < 0.005). The frequency of occurrence of each pathogen/disease was measured on a 0–5 scale (1 very infrequent, 5 very frequent, and 0 never observed). N/A: No answer.

**Table 4:** Results from the variables used in the analysis about the frequency of occurrence of the main pathogens in the nursery phase (%).

Differences	Pathogen	Variable	0	1	2	3	4	5	Sign.
Between consultants and field veterinarians	*M. hyopneumoniae*	Con.	36.4	0.0	18.2	45.5	0.0	0.0	0.049 *
		Vet.	14.6	27.0	24.7	18.0	14.6	1.1	
	PED	Con.	18.2	9.1	54.5	18.2	0.0	0.0	0.031 *
		Vet.	32.2	37.9	14.9	9.2	1.1	4.6	
	*C. perfringens type A*	Con.	45.5	18.2	0.0	18.2	18.2	0.0	0.006 *
		Vet.	44.2	31.4	18.6	4.7	1.2	0.0	
	*C. difficile*	Con.	54.5	18.2	9.1	18.2	0.0	0.0	0.021 *
		Vet.	56.5	32.9	9.4	1.2	0.0	0.0	
Between autonomous communities	SIV	A and C	20.8	37.5	10.4	25.0	6.3	0.0	0.017 *
		Rest	16.7	21.4	26.2	11.9	23.8	0.0	
	*L. intracellularis*	A and C	38.8	30.6	8.2	12.2	10.2	0.0	0.021 *
		Rest	35.7	45.2	16.7	0.0	0.0	2.4	
	PED	A and C	42.6	36.2	8.5	10.6	0.0	2.1	0.044 *
		Rest	16.7	35.7	26.2	11.9	2.4	7.1	

Con.: Consultants; Vet.: Field veterinarians; Sign.: Significant differences (* *p*-value < 0.05). The frequency of occurrence of each pathogen/disease was measured on a 0–5 scale (1 very infrequent, 5 very frequent, and 0 never observed). N/A: No answer.

**Table 5:** Results from the variables used in the analysis about the frequency of occurrence of the main pathogens in the fattening phase (%).

Differences	Pathogen	Variable	0	1	2	3	4	5	Sign.
Between consultants and field veterinarians	*C. perfringens type C*	Con.	50.0	0.0	16.7	16.7	16.7	0.0	0.017 *
		Vet.	37.2	33.3	11.5	16.7	1.3	0.0	
Between autonomous communities	SIV	A and C	17.1	24.4	31.7	22.0	4.9	0.0	0.045 *
		Rest	13.5	10.8	29.7	16.2	29.7	0.0	
	*L. intracellularis*	A and C	9.3	7.0	32.6	25.6	11.6	14.0	0.006 *
		Rest	5.4	18.9	37.8	0.0	29.7	8.1	
	*G. parasuis*	A and C	34.9	39.5	23.3	2.3	0.0	0.0	0.008 *
		Rest	13.5	35.1	21.6	18.9	10.8	0.0	
	PED	A and C	60.5	23.3	14.0	2.3	0.0	0.0	0.002 **
		Rest	21.6	24.3	45.9	2.7	5.4	0.0	

Con.: Consultants; Vet.: Field veterinarians; Sign.: Significant differences (* *p*-value < 0.05; ** *p*-value < 0.005).

## Data Availability

The datasets and materials used and/or analyzed during the current study are available from the corresponding author on request.
